# A randomized controlled trial of a group acceptance-based intervention for cancer survivors experiencing anxiety at re-entry (‘Valued Living’): study protocol

**DOI:** 10.1186/s12885-019-5289-x

**Published:** 2019-01-18

**Authors:** Joanna J. Arch, Jill L. Mitchell, Sarah R. Genung, Robert Fisher, David J. Andorsky, Annette L. Stanton

**Affiliations:** 1 Department of Psychology and Neuroscience, University of Colorado Boulder, 345 UCB Muenzinger, Boulder, CO 80309-0345 USA; 2 Division of Cancer Prevention and Control, University of Colorado Cancer Center, Aurora, CO 80045 USA; 3 Rocky Mountain Cancer Centers, Greenwood Village, CO 80111 USA; 4 Department of Psychology, University of California Los Angeles, Los Angeles, CA 90095 USA; 5 Department of Psychiatry and Biobehavioral Sciences, University of California Los Angeles, Los Angeles, CA 90095 USA; 6 Jonsson Comprehensive Cancer Center, University of California Los Angeles, Los Angeles, CA 90095 USA; 7 Cousins Center for Psychoneuroimmunology, UCLA Semel Institute for Neuroscience and Human Behavior, Los Angeles, CA 90095 USA

**Keywords:** Anxiety, Cancer, Cancer survivorship, Acceptance and commitment therapy, Acceptance, Psycho-oncology, Psychosocial intervention, Supportive care

## Abstract

**Background:**

Anxiety is a common concern of cancer survivors during the transition from active cancer treatment to cancer survivorship (the re-entry phase). This paper presents the study protocol for a novel group-based behavioral intervention to improve mental health, well-being, and medical use outcomes among anxious cancer survivors at re-entry.

**Methods/Design:**

This two-armed, prospective randomized controlled trial will randomize a minimum of 100 re-entry-phase cancer survivors with moderate to high anxiety to the intervention or a usual care control condition. The intervention is delivered in a group format over 7 weeks; content is based on Acceptance and Commitment Therapy (ACT), an acceptance, mindfulness, and values-based intervention. Participants will be recruited from community cancer care centers and the intervention will be led by the onsite clinical social workers. Participants will be assessed at baseline, mid-intervention, post-intervention, and 3- and 6-month follow-up. ACT participants will complete process measures before the beginning of group sessions 2, 4, and 6; all participants will complete the process measures during the regular assessments. The primary outcome is anxiety symptoms; secondary outcomes include anxiety disorder severity, fear of recurrence, depressive symptoms, cancer-related trauma symptoms, sense of life meaning, vitality/fatigue, and medical utilization.

**Discussion:**

This clinical trial will provide valuable evidence regarding the efficacy of the group ACT intervention in community oncology settings.

**Trial registration:**

Clinicaltrials.gov NCT02550925.

## Background

In the United States alone, there are over 15 million cancer survivors, representing nearly 5% of the total population. This number is projected to increase to over 20 million cancer survivors by 2026 [1]. A significant minority of cancer survivors report elevated levels of distress (see [2, 3]). Among various sources of distress, multiple studies suggest that one of the most common psychological concerns among cancer survivors is anxiety [3–6]. Fear of cancer recurrence is the most frequently cited source of anxiety, but the focus of anxiety extends beyond it to encompass additional domains, including anxiety about the effects of treatment and the ability to function in the face of enduring side effects, existential threats to one’s identity or sense of security, financial anxieties in the face of mounting medical bills, fear of investing in the future, and so forth [6, 7]. This study will assess the efficacy of a novel, theory-driven group intervention designed to address the psychological needs of anxious cancer survivors during the transition from cancer patient to post-treatment survivor (the re-entry phase)*.*

Anxiety can be particularly intense during the re-entry phase [6, 8, 9]. At re-entry, cancer survivors prone to anxiety may experience uncertainty about the meaning and purpose of their lives following cancer. Additionally they may worry: ‘Does this symptom mean that my cancer is back?’, ‘How can I live knowing that my cancer might return?’, or ‘Now that treatment is over, will my doctor abandon me?’ [6, 7, 9].

Elevated anxiety predicts the overutilization of oncology care [10] and primary care [11, 12], as well as dose delays or reductions of chemotherapy [13], lower quality of life [14–17], and possibly breast cancer recurrence [18] and earlier death following recurrence [19], though the latter findings are mixed. More broadly, elevated anxiety is linked to elevated risk for full-blown anxiety disorders [20], major depression and depressive symptoms [21], and fatigue [22]. A core symptom of anxiety disorders and elevated anxiety is behavioral avoidance of former activities and associated places, and intrusive or uncontrollable worry [23], each of which can diminish sources of daily meaning such as engagement with social, work, and family life.

For a large minority of cancer survivors, elevated anxiety persists for a decade or more following cancer treatment [3, 4], representing the largest psychological difference between long-term cancer survivors and controls [3, 24–26]. Addressing anxiety early in cancer survivorship—at re-entry, as presently proposed—thus serves a dual purpose: to ease acute anxiety during the often-challenging re-entry phase and to prevent the development of chronic, debilitating, and costly anxiety [3, 27, 28].

### Studies to date

The few prior anxiety-related interventions have focused on fear of recurrence [29–31] or uncertainty in later survivorship phases [32]. These interventions have taken a cognitive behavioral (CBT) or acceptance-based behavioral approach; randomized trials have shown that they significantly reduce fear of cancer recurrence relative to usual care [33], relaxation training [34], and supportive-expressive group therapy [29, 31]. Thus, CBT and related behavioral approaches have a successful track record of reducing fear of cancer recurrence.

Strong evidence demonstrates that cancer patients with higher levels of anxiety and distress benefit most substantially from psychosocial interventions, relative to their less distressed counterparts [35–37]. Yet except for a single study targeting distressed re-entry phase cancer survivors [38], the interventions developed for re-entry phase cancer survivors [39–48] target general cancer survivors rather than the anxious or distressed survivors most in need of help. This study represents one of the first interventions to focus on cancer survivors who report significant anxiety at reentry. By directing resources towards the cancer survivors most in need of support, and by focusing on the psychological symptom (anxiety) that is most likely to become chronic [3–5], the current approach represents an efficient use of psychosocial resources.

### The current intervention approach

The current intervention is founded on a theory-driven, evidence-based behavioral approach, Acceptance and Commitment Therapy (ACT), with demonstrated efficacy for treating anxiety [49–53]. In keeping with the ACT model [54–56], the intervention teaches anxious cancer survivors to: i) cultivate awareness of avoided thoughts and emotions about cancer; ii) disentangle from rigid, limiting thoughts and beliefs about themselves and their experience of cancer; and iii) commit to pursue meaningful life activities aligned with personally held values. According to the ACT model, these processes increase psychological flexibility – the capacity to focus on present-moment experience and to pursue personally valued life directions, even in the presence of internal discomfort (e.g., challenging emotions, physical pain, negative memories) [55–57].

ACT represents a potentially important paradigm shift for addressing anxiety and broader distress among cancer patients and survivors in that it allows for rather than minimizes the distress of cancer and fear of recurrence—an approach that may better validate the fears of re-entry phase cancer survivors, many of whom live with the real possibility of relapse and early mortality. In addition, beyond working to alleviate anxiety and fear of recurrence, ACT helps patients to explore personal values and sources of meaning, addressing the broad existential concerns of many cancer survivors [7, 58] and motivating movement in meaningful life directions.

Our pilot study for anxious cancer survivors at re-entry showed that relative to a one-month baseline period, ACT led to substantially reduced anxiety, depressive symptoms, and fear of recurrence, as well as increased vitality (i.e., lower fatigue) and sense of life meaning [59]. ACT thus helped anxious cancer survivors to increase their capacity to live meaningfully and effectively even *with* side effects and uncertainty about the future. Paradoxically, validating and actively accepting cancer-related distress also reduces it, as shown in this pilot study and in ACT studies with cancer patients during treatment [59–61]. Although not all trials have shown differences between ACT and traditional cognitive behavioral therapy (CBT) [62], a randomized trial for women with late-stage ovarian cancer showed that ACT, despite its lack of focus on symptom reduction, led to lower anxiety and higher quality of life than CBT by large and significant effect sizes [61].

We designed and piloted the current ACT intervention, known as “Valued Living for Cancer Survivors”, in close collaboration with community oncologists and social workers to meet the needs of community-based cancer clinics. First, rather than focus narrowly on a single diagnostic group, Valued Living includes patients across cancer type, an approach that is more sustainable and scalable, according to our community partners. In contrast, most psycho-oncology interventions target a single cancer type or cluster (e.g., breast cancer survivors, colorectal cancer survivors), that is unsustainable for care settings that lack the resources or participants to sustain distinct, intensive interventions for each major cancer type. Second, Valued Living intervenes at the group rather than individual level, representing an efficient use of psycho-oncology resources. Third, the Valued Living group facilitator manual was designed for efficient implementation by community oncology social workers by limiting the number of sessions and by focusing on a few core metaphors, thus enhancing dissemination potential even for psychosocial oncology professionals with limited ACT training. Fourth, the Valued Living groups are led by onsite oncology clinical social workers by leveraging part of their current role (to lead cancer support groups) in the study (to lead Valued Living groups). By contrast, many psycho-oncology interventions are designed and implemented using models or staffing that may not be sustainable in community settings. In that up to 85% of U.S. cancer patients are treated in the community, this approach aims to produce a protocol designed for dissemination in the community settings where the majority of cancer patients receive care.

### The current study

This study will assess the efficacy of Valued Living, a well-piloted ACT group intervention designed to address the psychological needs of anxious cancer survivors during the transition from cancer patient to post-treatment survivor (the re-entry phase). As noted, this trial addresses a number of limitations from previous trials in re-entry phase cancer survivors, first, by targeting anxious cancer survivors rather than cancer survivors generally; second, by adapting an evidence-based behavioral intervention model (ACT) to address the psychosocial needs of anxious cancer survivors; third, by collaboratively designing the intervention with community providers for sustainability in community-based cancer care settings.

Valued Living will be compared to an enhanced usual care control (UC)*.* This study thus aims to determine whether ACT improves outcomes for anxious cancer survivors beyond any benefits realized by usual care. More specifically, we will evaluate the hypothesis that ACT will improve psychosocial functioning by reducing negative effects such as anxiety symptoms (primary outcome), anxiety disorders, intrusive impact of cancer, fear of recurrence, and depressive symptoms, and by increasing positive effects such as appropriate medical care utilization, sense of life meaning, and vitality, relative to the UC control group. Second, to better understand how the group ACT intervention works, we will explore the active therapeutic processes that predict subsequent improvement in outcomes, including change in experiential avoidance and values-driven behavior.

## Methods/Design

### Study design

This two-armed, prospective randomized controlled trial (RCT) will randomize a minimum of 100 anxious re-entry-phase cancer survivors to group ACT (the Valued Living intervention) or a UC control condition. Participants will be randomized on a 1:1 ratio to ACT or UC using a computer-generated block randomization sequence done by a consulting biostatistician who is otherwise uninvolved in the study. All participants will be assessed at 5 points: baseline (Pre), mid-intervention (Mid), post-intervention (Post), and 3- and 6-month follow-up (3 m and 6 m FU). The Post assessment will occur 1 week after the final group session and the 3 m and 6 m FU will occur 3 and 6 months after Post. In addition, ACT participants will complete process measures before the beginning of group sessions 2, 4, and 6; all participants will complete the process measures during the regular assessments as well.

We considered alternative study designs such as comparing Valued Living to a more active condition, including an alternative psychosocial intervention or single educational session. However, in that this study reflects the first randomized trial of Valued Living, comparison to a staff-demanding condition or one that had not yet been empirically evaluated in the community oncology setting of the study, appeared premature.

### Study eligibility criteria

#### Inclusion criteria include

1. Adults (age 21+) in the Greater Boulder/ Denver, Colorado area who had cancer and completed primary cancer treatment (surgery, chemotherapy, and/or radiation) at minimum 6 weeks and maximum 24 months before the first group meeting, which approximates the ‘re-entry’ phase of cancer survivorship [8, 63].
2. For solid tumor cancers: Show no current evidence of disease for solid-tumor cancers (of any cancer type or initial stage). For lymphoproliferative disorders such as chronic lymphocytic leukemia and indolent non-Hodgkin’s lymphoma: The cancer is in remission or asymptomatic after initial treatment and patients are followed with active surveillance (“watchful waiting”).
3. Screen positive for anxiety symptoms on the study screener (see below)
4. Able to speak, read, and write English fluently
5. Willing and able to participate in the study, including completing online questionnaires and phone interviews at designated assessment points and if randomized, completing a group once a week for 7 weeks

#### Exclusion criteria include

1. Current moderate to high suicide risk
2. Psychiatric hospitalization or suicide attempt in the past 5 years
3. History of chronic untreated trauma (unrelated to cancer)

### Patient recruitment and consent

Cancer survivors will be recruited primarily from the greater Denver and Boulder metropolitan area offices of Rocky Mountain Cancer Centers (RMCC), the largest network of community oncology practices in Colorado. Patients will be recruited by flyers, targeted mailings, select local media ads, and referrals from survivorship visits with RMCC social workers, advanced practice providers, and medical oncologists. RMCC social workers will consent interested patients to have their contact information released to the University of Colorado research team or will have patients contact the research team directly. With these patients as well as those who self-refer in response to study flyers or media postings, the University of Colorado research team will conduct a final screening to assess all eligibility criteria and explain the details of study participation. Non-RMCC patients, if they learn about the study through a flyer or media posting, will also be eligible and will contact the research team directly to learn more about the study and for eligibility screening. RMCC medical providers will not consent patients to the study.

Following eligibility screening, the research team will send interested, eligible patients a written informed consent form. Patients will be encouraged to read it carefully, given the opportunity to review the consent form and address study-related questions with the research team, and given adequate time to consider whether they would like to voluntarily participate.

### Study conditions

As noted, participants will be randomized to either UC or ACT (the Valued Living intervention) on a 1:1 ratio.

#### Usual care control

Usual Care (UC) will consist of mailing each patient a list of oncology support group resources specifically tailored to each geographic area of recruitment. UC participants will also be encouraged to contact their RMCC social worker (or if they are not RMCC patients, the social worker at their cancer care center) for individual support as needed. UC is designed to provide ethical care for anxious cancer survivors using standard social worker time and resources. In both conditions, we will ask participants to report use of outside supportive and mental health resources on their medical trackers (see below) to account for their use in the analyses.

After the 6-month follow-up (i.e., the final assessment point), UC control participants will be offered the opportunity to participate in an ACT group (free of charge), thus maximizing potential study benefits for all patients. The data from these additional groups will not be included in the primary analyses because we risk confounding the data by comparing cancer survivors at different time points from re-entry and screening, and because we expect that not all UC participants will elect to complete the ACT group. By offering ACT to UC control participants as a benefit of follow-up completion, we aim to enhance patient care without confounding the data.

#### Valued living intervention

The ACT group intervention known as “Valued Living” will consist of 7 weekly group sessions of 2 h each. The Valued Living groups will meet at participating RMCC offices in group rooms appropriate for this purpose. In addition, participants randomized to Valued Living will be mailed the same list of geographically-tailored oncology support group resources that are mailed to participants randomized to usual care.

The Valued Living intervention is based on a facilitator manual and accompanying participant workbook developed during our single-arm pilot study [59]. In the pilot study, we developed and refined a flexible Valued Living manual based on a multi-step process of soliciting feedback from participants, social work group facilitators, and outside ACT experts. In response to their collective feedback, we iteratively refined the manual, including the overall intervention length, session length, and content.

During the first few group sessions of Valued Living, we introduce and use the Matrix [64], a simple tool to teach core ACT skills including increasing awareness of the link between internal experience and external behavior, personal values and their expression in daily life, and various forms of avoidance and their workability in the short- and long-term. In completing the Matrix, we ask group members to discuss their challenging thoughts and feelings related to cancer and the actions they take to get rid of or escape those feelings (struggle actions). We next have them share who and what are most important to them (values) and what they do when they feel connected to their values (valued actions). Whenever challenging cancer-related thoughts and feelings arise, we have them track their responses, and specifically what they do that moves them towards versus away from their values (or a mix of both).

In the middle Valued Living sessions, participants learn how to respond skillfully to cancer-related concerns and feelings and move towards valued actions, rather than remain stuck in struggle actions. A central tool in these sessions is the Passengers on the Bus metaphor [55], which invites participants to identify persistently challenging thoughts and images about cancer, labeled ‘passengers’. We physically enact this metaphor in the group. In doing so, we practice how to actively accept, and flexibly and compassionately relate to passengers while reducing their dominance over participants’ lives.

The final sessions of Valued Living focus more intensely on helping participants clarify their own values and a sense of purpose beyond, or even in the midst of, the threat of cancer. Participants also then focus more on committing to behaviors in alignment with those values, and on cultivating compassion for themselves in that process.

### Intervention training and facilitation

Participating RMCC offices have onsite clinical social workers who have expressed interest in learning ACT. Authors J.J.A. and J.L.M. train them in the ACT approach and the Valued Living protocol over 3 days, with 1 day of training every ~ 2 weeks to allow for readings and home practice between training days and to accommodate their clinical schedules. The training days incorporate active role-playing, experiential exercises, and coaching, followed by weekly feedback-based supervision, approaches recommended by the empirical literature [65, 66]. Second, an RMCC clinical social worker experienced in ACT co-facilitates the Valued Living groups, providing additional mentoring and consistency in the delivery of the intervention.

### Study ethics and integrity

Ethics approval was received from the University of Colorado Boulder Institutional Review Board (IRB) and Rocky Mountain Cancer Centers (RMCC); the University of Colorado Cancer Center approved the study protocol. Written informed consent will be obtained from all study participants. The University of Colorado has the right to audit the study. Protocol modifications will be submitted to the University of Colorado Boulder IRB and the University of Colorado Cancer Center. This is the second protocol version submitted to clinicaltrials.gov, dated July 27, 2018, reflecting the addition of anxiety disorder severity as a secondary outcome. Given the modest sample size and scope of the study, and the low-risk nature of the intervention, the study team did not elect to have a data monitoring committee. Any adverse study events will be reported to the University of Colorado Boulder IRB in a timely manner.

All participants will be assigned and referred to by their participant ID number to protect their identities. Participants’ names and ID numbers will only be linked in one secure, password-protected document stored separately from their demographic information or study data. Data will be entered into secure, password-protected databases stored on an encrypted external hard-drive in a locked research office. A separate research team member will verify all hand-entered data and check for participant entry errors for Qualtrics-collected data to help ensure the accuracy of the data. Participants will complete the baseline assessment before randomization to study condition. The principal investigator (PI), data analysts, and research team members conducting the outcome assessments will remain blind to condition assignment. Blindness for these team members will be achieved by not discussing condition status with the unblinded study coordinator, not attending the Valued Living groups, not rating videotaped sessions for fidelity (see below) until after the final assessment is conducted, and requesting during diagnostic assessment interviews that participants not reveal their assigned condition (see Anxiety Disorder measures, below). The CONSORT statement guidelines [67] inform the trial design throughout.

To implement the computer-generated block randomization sequence (see Study Design), the unblinded study coordinator will assign participant ID numbers in the consecutive order in which participants consent, and then link the list of ID numbers for each cohort to a spreadsheet column next to a hidden column of the block randomization sequence. The unblinded study coordinator then will unhide the sequence to determine which participant IDs are allocated to each condition in the ID sequence. The unblended coordinator will communicate condition assignment to participants and will videotape the group sessions and schedule the groups with the onsite social workers. Research team members experienced in ACT, none of whom run the Valued Living groups, will rate 2 randomly selected videos per group (~ 30% of sessions) for fidelity to the manual. They will rate only after extensive training to achieve respectable inter-rater reliability.

The PI can become unblinded to a participant’s study condition by the unblinded study coordinator in order to assess for potential clinical deterioration or emergency, such as in the case of patient-reported or indicated suicidality (assessed in the diagnostic interview, see Anxiety Disorder measures). Participants also can be withdrawn from the study at the PI’s discretion for reasons such as arriving to a group session inebriated or high, or exhibiting disruptive behaviors in group, e.g., yelling at or shaming other group members. To account for such occasional cases, participants will generally be discussed on the basis of their participant ID numbers for study purposes and will be discussed by first name in cases of potential clinical deterioration or study disruption; thus, if the name of a participant is revealed for purposes of PI assessment, then the PI will still remain blind to the participant’s ID number in the study database.

Study results will be communicated by submission to a peer-reviewed journal for publication and reporting of results in clinicaltrials.gov.

### Measures

As noted, outcome measures will be collected at 5 assessment points: Pre, Mid, Post, 3 m and 6 m FU. Given the time-intensive nature of the diagnostic interviews for participants, they will be collected at Pre, Post, and 6 m FU only. ACT process measures will be administered at Sessions 2, 4, and 6 of the intervention. Self-reported outcomes will be programmed and administered online in Qualtrics to ensure accurate data collection (or post mail if a participant lacks internet access). To encourage completion of the outcome measures, participants will be paid $25 per assessment point for measure completion, $25 per diagnostic interview completed, and $50 for a completed medical tracker (see Medical Utilization). Starting mid-way through the study, to encourage more timely measure completion, we began paying participants a $10 bonus for completing the questionnaires within 36 h of their receipt. Participants who drop out of the study will be given the opportunity to complete the study assessments. Diagnostic interviews will be conducted by phone with well-trained assessors who are blind to condition assignment (see Anxiety Disorders/ Diagnostic Interview). Cancer type and stage will be verified whenever possible through medical chart review.

#### Screening measure

The screener aims to identify cancer survivors who are notably anxious about cancer and anxious or depressed in their daily lives. Thus, anxious cancer survivors are eligible even if they also endorse depressive symptoms, reflecting the finding that anxiety and depressive symptoms frequently co-occur among cancer survivors [68] and more generally [69]. The 11 screening items include: 1) a validated 6-item version of the *State-Trait Anxiety Inventory* (STAI; [70]) that inquires about anxiety over the past month. The STAI is the most commonly used measure to identify which cancer patients benefit most from psychosocial interventions [35]. 2) The 4-item *Patient Health Questionnaire* (PHQ-4; [71]) brief measures of anxiety and depression symptoms, selected for brevity, ease of scoring, and consistency with the most recent American Society of Clinical Oncology distress screening guidelines [72]. 3) A 0–10 rating of “your current anxiety about cancer or the effects of cancer treatment”. Based on the Valued Living pilot study [59] and published validation studies, we will use the following as the cutoff for study eligibility: (1) a 5+ on the “anxiety about cancer” scale, which indicates moderate to severe anxiety about cancer *and* (2) a score of 14+ on the brief STAI [73] *or* 3+ on either the anxiety or depression scale of the PHQ-4 [71], which indicates significant anxiety or depressive symptoms in daily life.

### Primary outcome

#### Anxiety symptoms

The validated, widely-used *Hospital Anxiety and Depression Scale* (HADS; [74]) anxiety subscale will serve as the primary outcome due to its focus on broad anxiety symptoms and its wide use to evaluate anxiety and distress among cancer patients and survivors (e.g., [75, 76]).

### Secondary outcomes

#### Fear of recurrence

The *Concerns about Recurrence Scale* (CARS; [77]) will assess fear of cancer recurrence, which we adapted for all cancer types by substituting “cancer” for any reference to a specific form of cancer (i.e., “breast cancer”). Per our pilot study [59], we will specifically use the 4-item overall fear of recurrence subscale (e.g., “How much time do you spend thinking about the possibility that your cancer could recur?”, “How afraid are you that your cancer may recur?”).

#### Depressive symptoms

The 20-item *Center for Epidemiologic Studies Depression Scale* [78] will be used to assess depressive symptoms. The CESD was developed for use in medical populations [79] and is widely used and validated within cancer populations [78, 80].

#### Cancer-related trauma symptoms

The *Revised Impact of Events Scale* [81] will assess cancer-related hyperarousal, behavioral avoidance, and intrusion-related symptoms, which are known to be elevated among cancer patients and survivors [82].

#### Vitality/fatigue

The widely-used 4-item *SF-36 Vitality Scale* [83] assesses vitality/fatigue levels. This measure has been validated as a measure of energy/fatigue levels in cancer populations [84].

#### Sense of life meaning

The *Functional Assessment of Chronic Illness Therapy Spiritual Well-Being Scale,* meaning/peace subscale [85] will assess sense of life meaning. This validated measure has been widely employed in studies with cancer patients and survivors (e.g., [86, 87]) to assess a sense of meaning and purpose that is not specific to any particular religious or spiritual orientation.

#### Anxiety disorders/ diagnostic interview

At Pre-, Post- and 6-month FU, we will assess the presence and severity of anxiety disorders using the Mini-International Neuropsychiatric Interview (MINI) for DSM-5 [88]. The MINI will be enhanced with detailed diagnostic questions for anxiety disorders used in previous anxiety disorder studies and a more detailed suicidality assessment (e.g., [89]). As a diagnostic interview approach, the MINI possesses good test-retest reliability (70% of kappas are above 90%) and adequate concordance with other widely-used diagnostic instruments such as the Structured Clinical Interview Diagnostic and the Composite International Diagnostic Interview [90]. Disorder severity will be assessed with the validated 0 to 8 clinical severity rating (CSR) scale for each DSM disorder detected at the clinical or sub-clinical level [91].

Professional research assistants and clinical psychology doctoral students will conduct the MINI interviews after 10 to 15 h of training, including co-rating three gold standard training interviews from an independent research group, and demonstrating diagnostic and CSR accuracy on two to three gold-standard, recorded interviews from our own laboratory, and being coached in real-time by an experienced interviewer for their first two live patient interviews. Diagnoses and CSR ratings will be reviewed in weekly supervision with a licensed clinical psychologist (J.J.A.). As noted above, interviewers and J.J.A. will be blind to study condition.

#### Medical utilization

The *Medical Tracker*, based on previous psycho-oncology studies that have tracked medical utilization [39, 92], has participants track their medical appointments as well as appointments related to mental health and well-being such as support group(s), counseling, and related resources. It tracks the date of the appointment, the reason for the visit, including whether this visit was “related to your experience with cancer?”, the type of provider, whether the visit was a routine/ regular appointment or for a problem, whether they attended the appointment, and if so, how many minutes the appointment lasted. The medical tracker has two purposes: 1) to track attendance at routine cancer-related appointments and initiation of problem-related oncology visits, which serve as secondary outcomes, and 2) to track use of non-study supportive care and mental health care to account for their use in the statistical analyses. A randomly selected 15 to 20% of RMCC patients enrolled in the study will be selected for medical chart review to confirm the information listed by the patient in the *Medical Tracker*. Please note that for ethical reasons, participants will be permitted to pursue psychosocial interventions and support outside of the study; however, we will request that they track this care in their Medical Trackers.

### Process measures

We will use two measures to assess two core therapeutic processes hypothesized in ACT: experiential avoidance (the reverse of openness and acceptance of internal experience) and values-driven behavioral change [57] The *Brief Experiential Avoidance Questionnaire* [93], based on the longer *Multidimensional Experiential Avoidance Questionnaire* (MEAQ; [94]), is a psychometrically robust measure of experiential avoidance, the tendency to avoid challenging internal experiences such as negative thoughts and feelings. The BEAQ holds very similar psychometric properties as the MEAQ, which is distinct from neuroticism and negative affect [95].

The Bull’s Eye Values Survey part 1 [96] is a validated measure of values-driven behavior that is sensitive to ACT treatment effects. The original Bull’s Eye part 1 visually assessed the degree to which participants perceive living in accordance with their personal values in 4 domains: health, relationships, work/education, and leisure. Based on pilot participant and provider feedback, we adapted these for the current study to represent the following 5 domains: health & self-care, family relationships, social relationships & community, work/education, and leisure. We will administer the Bull’s Eye and BEAQ process measures at the beginning of group sessions 2, 4, and 6 using participant ID numbers rather than personal identifiers, and sealed envelopes to protect confidentiality, as well as during the full assessments in Qualtrics at baseline, post, 3- and 6-month FU.

### Power and sample size estimation

To estimate power for the hypothesized effects and to allow for the possibility of missing data in that estimation, we took a multi-faceted approach involving both existing software for power calculation in longitudinal multilevel linear modeling (PINT; [97, 98]) as well as a cutting-edge simulation approach (see [99, 100]). We started with hypothesized means on the main outcome (anxiety) for the treatment group and controls at each of four time points (baseline, post, 3- and 6-month FU). For the treatment group, these hypothesized means come from our pilot data [59]; the UC control group means represent a conservative expectation based on existing data from published studies [101]. Both sets of means show declines (linear and quadratic) over time, with weaker time effects for the control participants. Model specification involved a multilievel linear mixed model, specifying linear and quadratic effects of time (differing by condition), with random intercepts and time slopes for participants within groups. Power was estimated both for mean condition differences at each point in time as well as the condition by linear time interaction. Given the conservative means and the variance components, the hypothesized effect sizes were medium (*d* ~ .50) [102].

With no missing data and sample sizes of *n* = 50 in each group, the power to detect a significant condition by linear time interaction was 90% or greater. The power to detect the mean difference at 3-month FU was 88%. Simulations and PINT power software produced the same results, confirming excellent levels of power.

We then allowed data to be missing at random as a function of time (20% missing at Post; and 25% missing at each 3- and 6-month FU) – a situation that simulations can model [99, 100]. Our estimation that at least 75% of the sample will complete the 6-month FU (i.e., assuming 25% missing data at the final assessment point) is conservative. Even with this level of missing data, the power to detect a significant mean group difference at each Post and FU remained 81% or greater.

### Data analytic approach

For Aim 1, we will use multilevel mixed modeling to compare the ACT versus UC control groups on the rate of linear and curvilinear improvement, and at each assessment point, to compare between-group means. We will conduct separate intent-to-treat and intervention completer (“protocol adherent”) analyses. The HADS-Anxiety will serve as the primary outcome, although the other outcomes will be analyzed as well.

For Aim 2, to evaluate the session-by-session measures of active acceptance and values-driven behavior, first, we will assess change slopes over time for each process measure. Then, using time-lagged multilevel models, we will assess the extent to which change in active acceptance predicts subsequent change in values-driven behavior and vice versa, to identify the relationship between these two hypothesized ACT processes over time during the intervention. We will also test whether session-by-session change in each process predicts outcomes at Post and FU. If both putative processes significantly change over time and predict outcomes, then using a multiple mediation framework [103], we will assess which ACT process predicts outcomes more robustly when both processes are compared directly, to evaluate which process primarily drives outcomes.

Each analysis will include the hypothesized predictors as well as potential confounding factors (e.g., use of supportive care external to the study, cancer recurrence, age, gender). We will also conduct exploratory moderator analyses through subgroup analyses and moderation models to identify who most benefitted within each condition.

## Discussion

This randomized clinical trial will evaluate a well-piloted group intervention for anxious cancer survivors at re-entry within community oncology clinics. The intervention targets broadly anxious cancer survivors, across cancer type, using an evidence-based intervention approach that addresses psychological symptoms and existential concerns, delivered in a sustainable manner in community cancer care settings. If successful, the intervention has the potential to ease acute anxiety during the often-challenging re-entry phase and to prevent the development of chronic, debilitating, and costly anxiety [3, 27, 28]. The Valued Living intervention thus aims to provide enduring benefits to anxious cancer survivors.

## Abbreviations

ACT: Acceptance and commitment therapy
BEAQ: Brief Experiential avoidance questionnaire
CARS: Concerns about recurrence scale
CBT: Cognitive behavioral therapy
CESD: Center for epidemiologic studies depression scale
FU: Follow-up
HADS: Hospital anxiety and depression scale
MEAQ: Multidimensional experiential avoidance questionnaire
MINI: Mini-international neuropsychiatric interview
PHQ-4: 4-item patient health questionnaire
RMCC: Rocky mountain cancer centers
STAI: State-trait anxiety inventory
